# Supplementary material for: An open-source, end-to-end workflow for multidimensional photoemission spectroscopy
Source: Sci Data. 2020 Dec 17;7:442. doi: 10.1038/s41597-020-00769-8 (PMC7746702; doi:10.1038/s41597-020-00769-8)
Supplement: Supplementary file 1 — Supplementary Information [file 41597_2020_769_MOESM1_ESM.pdf]

Supplementary Information

An open-source, end-to-end workflow for multidimensional photoemission spectroscopy

R. Patrick Xian[1*], Y. Acremann[2], Steinn Y. Agustsson[3], Maciej Dendzik[1], Kevin Bühlmann[2], Davide Curcio[4], Dmytro Kutnyakhov[5,6], Federico Pressacco[6], Michael Heber[5], Shuo Dong[1], Tommaso Pincelli[1], Jure Demsar[3], Wilfried Wurth[5,6,†], Philip Hofmann[4], Martin Wolf[1], Markus Scheidgen[1,7], Laurenz Rettig[1*], and Ralph Ernstorfer[1*]

[1] Fritz Haber Institute of the Max Planck Society, 14195 Berlin, Germany
[2] Laboratory for Solid State Physics, ETH Zurich, 8093 Zurich, Switzerland
[3] Institute of Physics, University of Mainz, 55128 Mainz, Germany
[4] Department of Physics and Astronomy, Interdisciplinary Nanoscience Center (iNANO), Aarhus University, 8000 Aarhus C, Denmark
[5] DESY Photon Science, 22607 Hamburg, Germany
[6] Department of Physics, University of Hamburg, 22761 Hamburg, Germany
[7] Department of Physics, Humboldt University of Berlin, 12489 Berlin, Germany
[†]Deceased
*Corresponding authors: xian@fhi-berlin.mpg.de, rettig@fhi-berlin.mpg.de, ernstorfer@fhi-berlin.mpg.de

Table of Contents

S1 Metadata parameters and descriptions......**2**
- **Supplementary Table 1** 2
- **Supplementary Table 2** 3
- **Supplementary Table 3** 4
- **Supplementary Table 4** 5

References **5**

S1 Metadata parameters and descriptions

Supplementary Tables 1-4 list categorized parameters currently enlisted (version 1.0) as metadata for data deposition and parsing by the experimental materials science section of the NOMAD database [1]. Each parameter contains a name, unit, description, and lineage (i.e. immediate parent in the parameter tree), as implemented in `parser-mpes` [2]. For each parameter entry, providing “None” means “not applicable to the particular experiment or quantity, unknown or undetermined during the period of experiment”.

Supplementary Table 1: List of current general parameters.

Name	Unit	Description
experiment_location	None	Name of the city and country the experiment took place
experiment_date	None	Start and end dates of the experiment
experiment_summary	None	Descriptive summary of the content of the experiment
institution	None	Name of the institution hosting the facility
facility	None	Name of the experimental facility (e.g. in an acronym)
beamline	None	Name of the beamline the experiment took place
source_pump	None	Name or model of the pump light source
source_probe	None	Name or model of the probe light source
equipment	None	Name or model of the equipment (e.g. in an acronym)
sample	None	Description of the sample used in the experiment
measurement_axis	None	Names of the axes in measurement hardware
physical_axis	None	Names of the axes in physical terms

Supplementary Table 2: List of current source parameters

Name	Unit	Description
pump_rep_rate	Hz	Repetition rate of the pump source
pump_pulse_duration	fs	Pulse duration of the pump source
pump_wavelength	nm	Center wavelength of the pump source
pump_spectrum	None	Spectrum of the pump source
pump_photon_energy	eV	Photon energy of the pump source
pump_size	mm^2	Full-width at half-maximum size of the pump source at or closest to the sample position
pump_fluence	mJ/mm^2	Fluence of the pump source at or closest to the sample position
pump_polarization	None	Polarization of the pump source
pump_bunch	None	Total bunch number of the pump source
probe_rep_rate	Hz	Repetition rate of the probe source
probe_pulse_duration	fs	Pulse duration of the probe source
probe_wavelength	nm	Center wavelength of the probe source
probe_spectrum	None	Spectrum of the probe source
probe_photon_energy	eV	Photon energy of the probe source
probe_size	mm^2	Full-width at half-maximum size of the probe source at or closest to the sample position
probe_fluence	mJ/mm^2	Fluence of the probe source at or closest to the sample position
probe_polarization	None	Polarization of the probe source
probe_bunch	None	Total bunch number of the probe source
temporal_resolution	fs	Full-width at half-maximum of the pump-probe cross-correlation function

Supplementary Table 3: List of current detector parameters

Name	Unit	Description
extractor_voltage	V	Voltage between the extractor and the sample
work_distance	mm	Distance between the sample and the detector entrance
lens_names	None	Set of names for the electron-optic lenses
lens_voltages	V	Set of electron-optic lens voltages
tof_distance	m	Drift distance of the time-of-flight tube
tof_voltage	V	Voltage applied to the time-of-flight tube
sample_bias	V	Voltage bias applied to sample
magnification	None	Detector magnification
detector_voltage	V	Voltage applied to detector
detector_type	None	Description of the detector type (e.g. ‘MCP’, ‘CCD’, ‘CMOS’, etc.)
sensor_size	mm	Size of each of the imaging sensor chip on the detector
sensor_count	None	Number of imaging sensor chips on the detector
sensor_pixel_size	µm	Pixel size of the imaging sensor chip on the detector
x_to_momentum	$Å^{-1}$	Pixel x axis to k_x momentum calibration
y_to_momentum	$Å^{-1}$	Pixel y axis to k_y momentum calibration
tof_to_energy	eV	Time-of-flight to energy calibration
stage_to_delay	fs	Translation stage delay to pump-probe delay calibration
other_converts	None	Conversion factor between other measured and physical axes
momentum_resolution	$Å^{-1}$	Momentum resolution of the detector
spatial_resolution	µm	Spatial resolution of the source
energy_resolution	eV	Energy resolution of the detector

Supplementary Table 4: List of current sample parameters.

Name	Unit	Description
sample_id	None	Identification number or signatures of the sample used
sample_state	None	Physical state of the sample (e.g. 'gas', 'liquid', 'solution', 'amorphous solid', 'vitreous solid', 'monocrystalline solid', 'polycrystalline solid', etc.)
sample_purity	None	Chemical purity of the sample
sample_surface_term	None	Surface termination of the sample (if crystalline)
sample_layer	None	Sample layer or bulk structure (e.g. 'bulk', 'monolayer', 'multilayer_2' for bilayer, 'multilayer_5' for pentalayer, 'heterostructure', etc.)
sample_stacking	None	Stacking order of the solid surface (if crystalline)
sample_space_group	None	Space group of the sample compound (if crystalline)
chem_formula	None	Chemical formula of the sample
chem_elements	None	Symbols of the chemical elements contained in the sample
chem_name	None	Full chemical name of the sample
chem_id_cas	None	CAS registry number of the sample's chemical content
sample_temp	K	Temperature of the sample at the time of measurement
sample_pressure	Pa	Pressure surrounding the sample at the time of measurement
growth_method	None	Sample growth method (e.g. 'solution evaporation', 'chemical vapor transfer, 'molecular beam epitaxy', etc.)
preparation_method	None	Sample preparation method (e.g. 'in-vacuum cleaving', 'ex-vacuum cleaving', 'ion milling', etc.)
sample_vendor	None	Name of the sample vendor (e.g. a company name or 'homemade')
substrate_material	None	Material of the substrate the sample has immediate contact with
substrate_state	None	State of matter of the substrate material (similar options as those in the sample_state parameter)
substrate_vendor	None	Name of the substrate vendor (similar to options in sample_vendor)

References

1. Draxl, C. & Scheffler, M. NOMAD: The FAIR concept for big data-driven materials science. *MRS Bulletin* **43**, 676-682 (2018).
2. parser-mpes, https://gitlab.mpcdf.mpg.de/rpx/parser-mpes
